# The efficacy of calcein as a tagging agent for the long-spined sea urchin, *Diadema antillarum*

**DOI:** 10.17912/micropub.biology.002242

**Published:** 2026-09-25

**Authors:** Benjamin A. Belgrad, Lindsay J. Spiers, William C. Sharp

**Affiliations:** 1 Fish and Wildlife Research Institute, Florida Fish and Wildlife Conservation Commission, Marathon, FL, United States; 2 Fish and Wildlife Research Institute, Florida Fish and Wildlife Conservation Commission

## Abstract

The sea urchin *Diadema antillarum* is a restoration target due to its function as a keystone reef herbivore. However, restocking efforts are hindered by a lack of long-term tagging methods. We tested the effectiveness of calcein marking for urchin releases. Cultured individuals were tagged using a 150 mg/L calcein bath and monitored for 11 weeks. Dye markings remained fully visible in all individuals for two weeks but began to fade within three weeks. Only individuals with white spines maintained markings beyond five weeks. Unlike with other urchin species, calcein only seems suitable for short-term external tagging of *D. antillarum* .

**Figure 1. Photographs of calcein tagged and control urchins and duration of tag visibility:** Photographs of the same two urchins tagged with calcein under **a)** normal light, **b)** unfiltered UV light, and **c)** UV light viewed through a blue light filter. Fluorescence from tagging was best viewed under blue filtered UV light and was observed on either **c)** the spines or **d)** tube feet along the ambulacral plates. **e)** Number of urchins with only black spines or with some white spines still exhibiting fluorescent tagging each week (n = 13 urchins tagged total). Urchins with black spines had fluorescence intensity notably decline and become harder to detect visually three weeks after tagging. **f)** Urchins with white spines that were either dyed with calcein (tagged) or undyed (control) 10 weeks after tagging. **g)** A black and white spine from a tagged and control urchin viewed under filtered UV light. **h–l)** Photo series spanning ~30 seconds of an urchin expelling fluorescent substance three days after tagging. Yellow arrow indicates initial material expulsion.

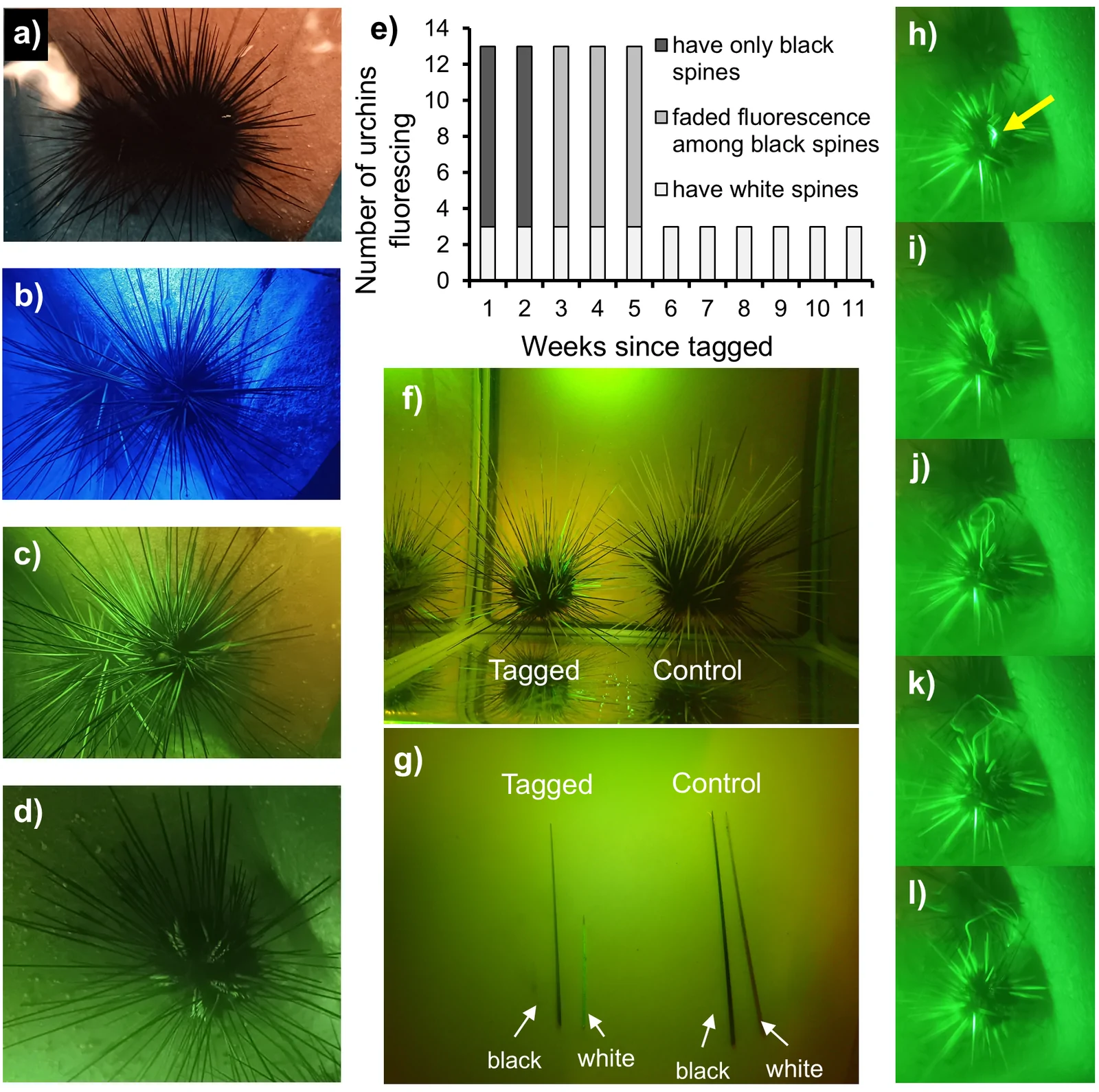



## Description

Sea urchins comprise a global multimillion dollar fishery and are ecological engineers (Ogden et al., 1978; Stefánsson et al., 2017; FAO 2025). As their populations have declined, urchins are increasingly cultivated around the world for consumption and restoration purposes (FAO 2024; Rakaj et al., 2024; Smith et al., 2026). The long-spined sea urchin, *Diadema antillarum* , is a keystone herbivore native to the tropical Atlantic and Caribbean coral reefs (Randall et al., 1963; Carpenter 1981; Carpenter 1988; Lessios 2016). In the 1980s, an epidemic spread throughout the Caribbean and killed 93–98% of the urchin populations (Carpenter 1988; Lessios 1988). This was followed by an enormous increase in benthic macroalgae and a decrease in coral cover as the coral was outcompeted for space and light (Hughes et al., 1987). *D. antillarum* populations have only modestly recovered since this epidemic (~12% population pre-epidemic; Lessios 2016), and the species has recently become a target of restoration to aid coral recovery (Pilnick et al., 2021; Hylkema et al., 2022). In areas where *D. antillarum* populations have increased in abundance, there have been significant decreases in macroalgal cover and, in some areas, increases in coral recruitment (Edmunds and Carpenter 2001). The restoration effort has centered around supplementing wild populations with hatchery reared juveniles (Pilnick et al., 2021; Hudspeth et al., 2026), as natural recruitment is low and early life-stage predation is high (Lessios 2016; Hylkema et al., 2022). However, restocking efforts and research on the effectiveness of this approach are hampered by a lack of viable long-term tagging methods. Reliable long-term tagging is essential to obtain information that is largely lacking and fundamental to developing and optimizing restoration strategies for this species, such as post-release survival, individual growth rates, and movement patterns of outplanted urchins.

Various methods of tagging urchins have been employed since the 1960s with mixed success (Ebert 1965; Ebert 2013; Santos et al., 2022; Tourón et al., 2023). Many internal physical tags such as decimal coded wire tags (CWTs) and passive integrated transponder (PIT) tags can cause increased mortality or decreased growth rates in urchins, especially in smaller individuals, while external physical markings such as painting spines or placing beads are quickly lost (Cipriano et al., 2014; Santos et al., 2022; Tourón et al., 2023). Chemically marking individuals with the fluorochrome dye calcein is a potential noninvasive and non-toxic means of tagging *D. antillarum* for long durations. The molecule typically binds irreversibly to calcium ions and becomes incorporated into the tissue and skeletal structure of animals as they grow, which causes the individual’s tissues to fluoresce under UV light (Johnson et al., 2013; Li et al., 2020). Although this method has been used to tag several species of urchins for months (Johnson et al., 2013; Rodríguez et al., 2016; Santos et al., 2022; Tourón et al., 2023), the technique has yet to be tested with *D. antillarum* . Here, we conducted a laboratory study to evaluate the efficacy of using calcein to tag *D. antillarum* for extended timeframes.

Survival was 100% over the course of this study. Recently tagged *D. antillarum* fluoresced strongly under UV light and the markings were most visible when observed through a blue light filter (Figure 1a–c). Individuals differed in where tag fluorescence appeared on their body as fluorescence was either localized to their spines (Figure 1c) or tube feet along the ambulacral plates (Figure 1d). Fluorescence on completely black urchins followed similar intensity levels and duration regardless of whether it occurred on spines or tube feet. Tag fluorescence started to become difficult to detect visually in black spined urchins after two weeks and was unlikely to be observed under SCUBA diving conditions beyond this point (Figure 1e). Most tagged urchins ( *n* = 10 of 13) were indistinguishable from untagged control urchins ( *n* = 21) after five weeks; however, all three urchins with white spines ( *n* = 3 of 13) retained fluorescence for at least 11 weeks (Figure 1f). This continued fluorescence was limited to the white spines (Figure 1g), possibly either because the darker pigmentation within black spines prevented UV light from reaching the incorporated calcein, or it may have attenuated emission visibility, masking calcein fluorescence. However, further molecular studies are needed to identify the mechanisms underlying differences in spine fluorescence. Interestingly, we observed four individuals periodically releasing a fluorescent substance aborally three days after removal from the dye bath. This may indicate post-exposure clearance or redistribution of dye-associated material, though chemical confirmation was not performed (Figure 1h–l).

Importantly, unlike previous studies where calcein dye was visible externally on urchins for months (Rogers-Bennett 2003; Johnson et al., 2013; Santos et al., 2022; Correia et al., 2023; Tourón et al., 2023), dye markings only remained visible on *D. antillarum* for approximately two weeks — despite our use of a more intense than typical dye regimen. Such contrasting results are likely due to differences in pigmentation of those species as *Paracentrotus lividus* (Santos et al., 2022), *Strongylocentrotus droebachiensis* (Johnson et al. 2013), *Strongylocentrotus franciscanus* (Rogers-Bennett 2003), and *Pseudechinus huttoni* (Kirby et al., 2006) are substantially lighter in coloration than *D. antillarum* . In the dark-colored *Diadema africanum* (Rodríguez et al., 2016) and *Arbacia lixula* (Barrera 2018), extended tag visibility was only achieved by dissection and microscopic examination of the Aristotle’s lantern. Consequently, we conclude that calcein dye is unsuitable for tagging *D. antillarum* for long durations when individual survival is a priority or disturbance must be minimized, but calcein dye may be sufficient for short-term studies or when dissection is an option. These results highlight how even biologically conserved chemical reactions can have different outcomes across similar species and the necessity for species-specific studies.

## Methods

Juvenile *Diadema antillarum* , spawned from wild-caught broodstock, were acquired from The Florida Aquarium's Center for Conservation, Apollo Beach, FL, US, in May 2024. Individuals were transported to the Florida Fish and Wildlife Research Institute’s South Florida Regional Lab in Marathon, FL, US, where they were nursed for approximately 21 months before commencing the experiment. During the nursery period, urchins were held in circular flow-through tanks (diameter = 155 cm, water height = 70 cm) under similar conditions to Sharp et al. (2023). Sand-filtered seawater was supplied from Florida Bay and matched natural abiotic conditions. Shelter was provided to urchins in the form of longitudinally halved terracotta pots (22 cm L × 22 cm W × 12 cm H) that mimicked natural coral reef crevice habitat, and urchins were fed satiating amounts of drift algae.

Thirty-four urchins were haphazardly selected for tagging ( *n* = 13; test diameter = 27–43 mm) and control treatments ( *n* = 21; test diameter = 32–61 mm) to verify that untagged urchins do not fluoresce and determine if tagging influences survival. Urchins selected for tagging were distributed across three continuously aerated tanks containing a solution of sand-filtered seawater with 150 mg/L concentration of dissolved calcein. Two tanks held five urchins with 11 L calcein solution (Chemcruz source) while the third tank held three urchins with 7 L calcein solution (MP Biomedicals source). These volumes and urchin numbers were chosen based on perceived physiological tolerances, and the compounds from both sources produced the same results. Urchins were immersed in this dye bath for seven days (typical dye regimens use 100–150 mg/L for 24–48 h immersion). This extended period of immersion was performed because our preliminary experiments noted that fluorescence tagging disappeared quickly, and we wanted to identify the maximum amount of time tagging can be viewed externally on pure black-spined urchins, which comprise the majority of *D. antillarum* populations.

 After this tagging period, control and tagged urchins were placed into two separate 356-L rectangular aquaria (120 cm L × 75 cm W × 36 cm H) and maintained under similar conditions as the nursery period. Individuals were held for 11 weeks and checked at least weekly to determine if fluorescent markings were still observable. Markings were observed by shining a Sola NIGHTSEA dive light (emission spectra 420–470 nm, peaking at ~445 nm) on individuals and viewing them through a NIGHTSEA blue blocking light filter visor (blocking wavelengths shorter than 500 nm).

## Reagents

**Table:** 

Reagent	Alternate names	Lot Number	Source
Calcein disodium salt	Fluorexon Sodium Salt	C2425	ChemCruz
Calcein	Fluorexon; Fluorescein-bis(methyliminodiacetic acid); Bis[ *N,N* −bis(carboxymethyl)aminomethyl]fluorescein; 3,3'-bis[ *N,N'* -di(carboxymethyl)-aminomethyl]-fluorescein; Calcein W	S8883-2	MP Biomedicals
